# The Significance of Topoisomerase II Alpha in Invasive Breast Carcinoma

**DOI:** 10.7759/cureus.18733

**Published:** 2021-10-13

**Authors:** M Arthi, K Arun Kumar, Lawrence D'Cruze, Rajendiran S, Leena D Joseph, Bhawna Dev

**Affiliations:** 1 Pathology, Sri Ramachandra Institute of Higher Education and Research, Chennai, IND; 2 General Surgery, Sri Ramachandra Institute of Higher Education and Research, Chennai, IND; 3 Radiology, Sri Ramachandra Institute of Higher Education and Research, Chennai, IND

**Keywords:** breast, top2a, prognosis, er, pr, her2neu, immunohistochemistry

## Abstract

Background

Topoisomerase II alpha (Top 2 A) protein has been shown to be a proliferation marker associated with tumour grade. The current study evaluated the prognostic impact of Top 2 A protein on luminal breast cancer and its utility as an independent prognostic marker. Immunohistochemical expression of Top 2 A in breast cancer and its correlation with the tumour type, size, lymph node metastases, grade and ER/PR positivity.

Methodology

Ethics committee approval was taken and 65 cases of Invasive breast carcinoma presenting to the Department of Pathology at a tertiary care centre in South India were studied. Patient details including age, tumour type, tumour size, tumour grading, estrogen receptor (ER)/progesterone receptor (PR)/human epidermal growth factor receptor 2 (HER2/neu) status and pathologic stage was studied. Immunohistochemistry (IHC) work-up for Top 2 A expression was done and evaluated.

Results

Of the 65 histological sections of breast cancers, 29/65 showed nuclear positivity for Top 2 A. Node positive tumours 17/65 stained positive for Top 2 A. Stage I tumours 2/65, stage II tumours 12/65 and stage III 14/65 stained positive for Top 2 A. Among the HER2/neu-positive tumours, 22/65 stained for Top 2 A and among ER/PR-positive 9/65 cases were positive for Top 2 A. Triple-negative tumours 5/65 stained for Top 2 A.

Conclusion

Higher Top 2 A expression was seen in higher stage tumours. HER2/neu-positive tumours significantly showed a correlation with Top 2 A positivity. Therefore, Top 2 A expression can be considered an individual prognostic factor in breast carcinoma.

## Introduction

Worldwide, breast cancer is the most frequently diagnosed life-threatening cancer in women. In less-developed countries, it is the leading cause of cancer death in women. In India, the average age of developing breast cancer has undergone a significant shift over the last few decades and is rapidly becoming one of the most common cancers in females pushing cervical cancer to second place and also accounts for a major cause of mortality and morbidity.

For many years the hormone receptors expression has been the prognostic and therapeutic marker in invasive breast cancer. Identification of human epidermal growth factor receptor 2 (HER2/neu) and development of the targeted therapy trastuzumab for those who overexpress it has been the most important milestone in the search for individualized therapy. New markers like topoisomerase II alpha have been proposed as a prognostic and also targeted therapy marker. Studies in this context are only limited and have shown varying results as predictors for disease outcome with this marker.

TOP 2 A gene encodes for topoisomerase 2 alpha (Top 2 A) which is a 170kd protein located at chromosome 17 is up-regulated by the proliferating cells. TOP 2 A is also a molecular target for some important anticancer drugs, including anthracyclines. Exclusive of Top 2 A potential role as a target for anticancer drugs, few studies have analysed it as a potential prognostic marker in breast cancer. Biologically it is unclear as to why HER2 status should influence co-amplification of Top 2 A sensitivity. Their genomic proximity on chromosome 17q12-q21 and frequent co-amplification in many breast cancer provide a biological rationale for such a notion. These strengths and limitations in HER2 and Top 2 A detection must be considered when exploring their potential role as predictive tools. 

The current study focuses on the association of Top 2 A expression with tumour grade, stage, molecular type and also its use as an independent prognostic marker. This study was conducted retrospectively on 65 cases of breast carcinoma, comprising of invasive breast carcinoma diagnosed at a tertiary care centre from 2011 to 2014 in the Department of Pathology.

Aims and objectives

The aims of this study are: (1) to evaluate the expression of Topoisomerase II Alpha in Invasive mammary carcinoma, (2) to evaluate the utility of Top 2 A as an independent prognostic marker, and (3) immunohistochemical (IHC) expression of Top 2 A in breast cancer and its correlation with the tumour type, size, pathologic stage, grade and estrogen receptor (ER)/progesterone receptor (PR) and HER2/neu status.

## Materials and methods

This is a retrospective study on archival material of paraffin blocks of 65 invasive carcinoma specimens received in the Department of Pathology at a tertiary care centre from 2011 to 2014.

Inclusion criteria

Microscopically proven modified radical mastectomy specimen with invasive breast carcinoma for which ER/PR and HER2/neu status is known.

Exclusion criteria

Core biopsy, excision biopsy and simple mastectomy specimens were excluded in order to know the TNM staging of the cancer. 

Ethical clearance for this study was obtained from the Institutional Ethics review committee.

Clinical details of patients including age, tumour type, tumour size, tumour grading using Nottingham grading system, ER/PR/ and HER2/neu status, metastasis and pathologic stage were obtained from the hospital records. Archived cases of invasive breast carcinoma were selected and blocks with sections containing normal and tumour were chosen for immunohistochemistry study.

This immunohistochemical study of topoisomerase II alpha (Clone: EP93/ Isotype: Rabbit IgG/ Source: Rabbit Monoclonal/Immunogen), a synthetic peptide corresponding to C-terminal residues of human topoisomerase II alpha was conducted on an estimated sample size of 65 cases of histopathologically proven invasive carcinoma

**Figure 1:**
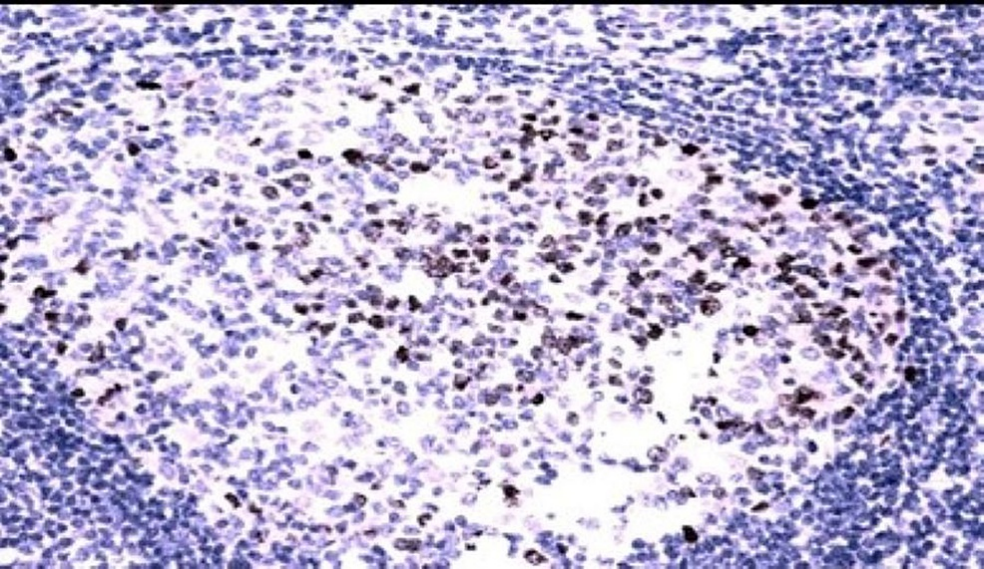
Top 2 A positive control Top 2 A positive control IHC 400X IHC: immunohistochemistry; Top 2 A: Topoisomerase II Alpha

Mitotic figures in tonsillar tissue are strongly positive for Top 2 A and this was taken as the positive control [Fig. 1].

As the negative control, the slide was treated by replacement of primary antibody with non-immune serum.

**Figure 2:**
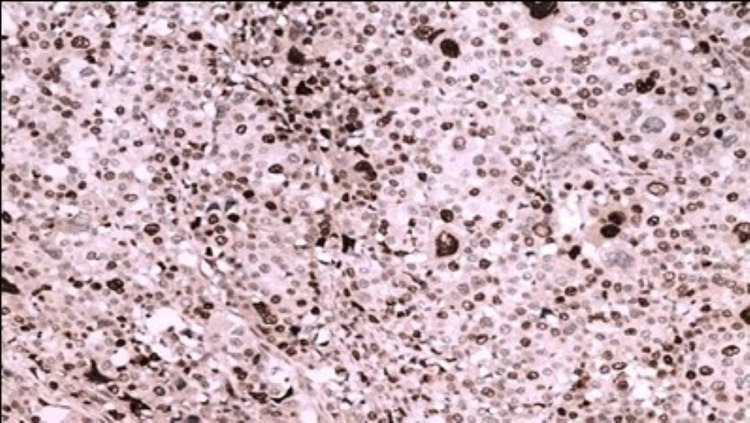
Top 2 A positive IHC 200X Top 2 A positivity in invasive ductal carcinoma IHC 200X IHC: immunohistochemistry; Top 2 A: Topoisomerase II Alpha

**Figure 3:**
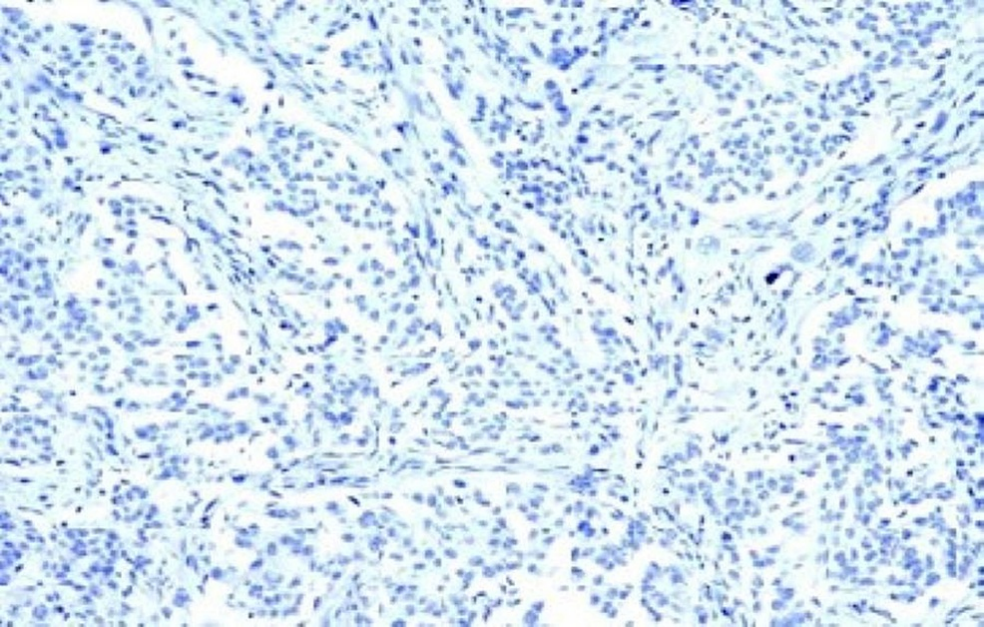
Top 2 A negative 200X IHC Top 2 A negative in invasive ductal carcinoma 200X IHC

All samples showing > 10% of tumour cells with positive nuclear staining were defined as topoisomerase II alpha positive [Fig. 2], while samples with < 10% of positive cells were defined as topoisomerase II alpha negative [1] [Fig. 3].

All cases of histopathologically proven Invasive carcinoma diagnosed in the Department of Pathology, at the tertiary care centre between 2011 and 2014 for which ER/PR and HER2/neu status was known were included in the study. A total of 65 cases were identified and studied.

Statistical analysis

Statistical analysis was done on the data collected by using the software GNU-PSPP version 0.10.1 (http://www.gnu.org/software/pspp). Pearson Chi-square test was used to determine significant clinicopathological differences between Top 2 A expression in positive and negative tumours. Differences were considered statistically significant when the p-value was < 0.05. 

## Results

Demographic details 

The age of study participants ranged from 26 to 80 years. The highest incidence was noticed in the age group of 51-60 years (33.8%). The second highest incidence was seen in the age group of 41 to 50 years (26.1%).

Tumour characteristics

In this study, the tumour size varied from 1.2 cm to 14 cm with a median tumour size of 3.5 cm. The highest incidence of the tumour was in the upper outer quadrant (33.7%). The next common site was the central quadrant (26.1%). The most common histological type of breast cancer was invasive ductal carcinoma (IDC) not otherwise specified (NOS) which constituted 58 (89.2%) of the study population. Invasive lobular carcinoma was the second common (9.2%). The other histological type was mucinous carcinoma breast (1.5%).

Grading and staging

Nottingham histological grade was done on all 65 cases of invasive breast carcinoma which showed 47.6% of grade III tumour, 46.1% of grade II tumour and 6.1% of grade I tumour.

Nottingham histologic grade

Among the 58 cases of invasive ductal carcinoma 28 (48.2%) cases were grade II followed by 27 (46.5%) of grade III and three (5.1%) cases of grade I. Among six cases of Invasive lobular carcinoma 4 (66.6%) cases were grade III, two (33.3%) were grade II and none in grade I. There was only one case of mucinous carcinoma which was grade I (100%). Amongst the 65 cases of invasive breast carcinoma, the most commonly reported tumour stage (pT) was stage II (58.4%) followed by stage III (24.6%), stage IV (9.2%) and stage I (7.6%). In Invasive ductal carcinoma, most of the cases were stage II (58.6%), stage III (24.1%), stage IV (10.3%) and stage I (6.8%). In invasive lobular carcinoma, most of the cases were stage II (50%), stage III (33.3%) and stage I (16.6%). One case of mucinous carcinoma breast showed stage II. It was noted in the study that low-grade tumours (grade I and II) were commonly associated with the low stage (stage I and II) and high-grade tumours (grade III) was associated with the high stage. A combination of low grade and low stage tumours was seen in 62.7% of cases and a combination of high grade and high stage tumours was seen in 72.7%. Nodal metastasis (pN1) was seen present in 38 (58.4%) and absent (pN0) in 27 (41.5%). Distant metastasis (cM1) was present in only three (4.6%) and absent (cM0) in 62 cases (95.3%).

Additional pathological findings

Among the 65 cases of invasive carcinoma breast, 24 (36.9%) cases showed carcinoma in situ components and 41 (63.08%) cases did not show any in situ changes. Lymphovascular invasion was present in 33 (50.75%) cases and absent in 32 (49.2%) cases.

Molecular classification (based on the result of ER/PR and HER2/neu by IHC)

Among the 65 cases of Invasive breast carcinoma studied ER/PR positivity was seen in 25 (38.4%) cases and negativity was observed in 40 (61.5%) cases. HER2/neu was positive in 52.3% of cases and negative in 47.69% of cases. The majority of the cases was HER2/neu type of mammary cancer 25 (38.4%) followed by Luminal A type in 14 (21.5%) of cases, Luminal B type in 11 (16.9%) cases and triple-negative in 15 (23.07%) cases.

Topoisomerase II alpha immunohistochemistry

Among the 65 cases of invasive breast carcinoma, 29 (44.6%) cases showed nuclear expression of topoisomerase II alpha and 36 (55.3%) cases were negative for topoisomerase II alpha. Immunohistochemical expression of topoisomerase II alpha did not show any significant correlation with increasing age (p=0.344)[Table 1]**.** Expression of topoisomerase II alpha was not significant with tumour size (p = 0.449) [Table 1]. Out of 58 cases of Invasive ductal carcinoma 23 (39.6%) showed topoisomerase II alpha expression and 35 cases (53.8%) were negative for topoisomerase II alpha. There was a significant statistical correlation seen between Invasive ductal carcinoma and topoisomerase II alpha expression (p= 0.017). 

**Table 1:** Comparison of Top 2 A with various parameters Top 2 A - Topoisomerase II Alpha; LVI - Lymphovascular invasion; DCIS - Ductal carcinoma in situ

Characteristics	Top II A status		p-value
	Positive n(%)	Negative n(%)	0.344
Age			
< 50	20(13)	18.4(12)	
>50	24.6(16)	36.9(24)	
Size			0.700
<5cm	47.8(11)	52.1(12)	
>5cm	42.8(18)	57.1(24)	
Nodal status			0.981
Positive	44.7(17)	55.2(21)	
Negative	44.4(29)	55.5(15)	
Grade			
I	40(2)	60(3)	0.828
II	43.3(12)	56.6(17)	0.847
III	45.1(14)	54.8(17)	0.933
LVI			0.173
Present	36.3(12)	63.6(21)	
Absent	53.1(17)	46.8(15)	
DCIS			0.052
Present	29.1(7)	70.8(17)	
Absent	53.6(22)	46.3(19)	

Relationship of Top 2 A with IDC

Out of six invasive lobular carcinoma five (83.3%) cases showed positivity for topoisomerase II alpha and one (16.6%) case was negative for topoisomerase II alpha with a significant correlational value of p=0.040. One case of mucinous carcinoma breast was positive for topoisomerase II alpha (100%). Expression of topoisomerase II alpha was found to be 40% for grade I (G1) tumours, 43.3% for grade II (G2) tumours and 45.16% for grade III (G3) tumours [Table 1]. There is no significant correlation between the grade of the tumour and topoisomerase II alpha expression (p-value = G1 - 0.603; G2 - 0.523; G3 - 0.565). Expression of topoisomerase II alpha in lymph node-positive cases was 44.7%. No significant correlation was seen (p=0.591) [Table 1]. Expression of topoisomerase II alpha in cases with lymph vascular invasion was 33.36%. No significant correlation was seen (p=0.134) [Table 1]. Topoisomerase II alpha expression in patients with carcinoma in situ changes were 29.1% and those without in situ changes were 70.8% [Table 1]. There was no significant correlation between Top 2 A expression and carcinoma in situ changes (p=0.052). Expression of topoisomerase II alpha in patients with ER/PR positive Invasive breast carcinoma was 36%. No significant correlation is seen (p=0.198). 

Luminal A type of breast cancer cases showed a topoisomerase II alpha expression in only 7.14% cases with a significant statistical correlation value of p=0.001 [Table 2]. Expression of topoisomerase II alpha in Luminal B molecular type of breast carcinoma was seen only in eight cases with a significant statistical correlation value of p=0.038 [Table 2]. HER2/neu type of invasive breast cancer showed topoisomerase II alpha positivity in 15 (60%) cases. There was a significant statistical correlation (p=0.048) [Table 2]. Among the 15 cases of the triple-negative type of breast carcinoma, only five (35.7%) showed topoisomerase II alpha expression. No statistical correlation was observed (p=0.446) [Table 2].

**Table 2:** Expression of Top 2 A in molecular types of aammary carcinoma Top 2 A - Topoisomerase II Alpha

Top 2 A	Luminal A type (n-14)	Luminal B type (n – 11)	HER2/neu (n-25)	Triple-negative (n-15)
Positive	1	8	15	5
Negative	13	3	10	10

## Discussion

The therapeutic modalities and prognosis of breast cancer are influenced by several factors. According to Cristofanilli M et al, prognostic factors for breast cancer include age, tumour size, histological subtype and grade, axillary lymph node status, lymphatic/vascular invasion, hormone receptor status [2].

In India, the average age of high risk is 43 to 46 years [3]. In our study, the age ranged from 26 years to 80 years with a median age of 52 years. The highest number of cases were seen between the age group of 51 to 60 years. This is in concurrence with the study by Youlden DR et al [3].

The most common histological type of breast cancer observed in our study was invasive ductal carcinoma followed by invasive lobular carcinoma. This accounts for 89.7% of IDC and 9.2% of lobular carcinoma. This is in concordance with Malhotra GK et al [4] who also observed similar findings.

Among the 65 cases studied, the size of the tumour varied from 1.2 cm to 14 cm with an average size of 3.5 cm. A similar observation was seen by Rody A et al [1]. Their study also showed that most of the patients were in the pT2 stage (44%) of tumour. Similarly, even our study showed the majority of the patient in the pT2 stage (58.4%).

Most of the studies showed Grade II tumours to be more common following Grade III and Grade I. In our study Grade III tumours were seen in 31 cases (47.6%) and Grade II tumours in 30 cases (46.1%).

In our study lymph node positivity was seen in 38 (58.4%) and nodal status negative in 27 (41.54%). In a study conducted by Depowski PL et al [5] in 89 cases, 40% showed node positivity.

El Rebey HS et al [6] in their study of 84 patients of Invasive breast carcinoma observed that carcinoma in situ changes was seen in 71.4% and absent in 28.6% of cases. Our present study also showed a similar observation; carcinoma in situ was absent in 63.08% and present in 36.9%.

The next important parameter which is essential for planning therapy and prediction of prognosis is ER, PR and HER2/neu hormonal status mainly. 

In most of the studies conducted in invasive breast carcinoma, the most common molecular positivity is with ER/PR, followed by HER2/neu and then triple-negatives [7,8]. In our study, HER2/neu was found to be the most common molecular marker amplified.

Considering the above study results and according to Onitilo AA et al [9]. Luminal A was the most common molecular type. But in our study, it is observed that HER2/neu type molecular type of breast cancer was the most common (38.6%).

Topoisomerase 2 alpha plays a key role in DNA replication and is a target for multiple chemotherapeutic agents. In breast cancer, Top 2 A expression has been linked to cell proliferation and HER2/neu protein overexpression. However, its relationship with outcome variables is not well established.

Studies have shown a significant association of increased expression of topoisomerase II alpha with known outcome variables. Of the few studies that have highlighted this association, most support our findings.

In our study, most of the patients fell under 51-60 years of age and no significant statistical correlation was seen between age and topoisomerase II alpha expression (p=0.344). This is in concordance with El Rebey HS et al [6], in which no statistical correlation was observed.

Out of the 65 cases studied, the tumour size varied from 1.2-14 cm with a mean size of 3.5 cm. In our study, no statistical correlation was seen between the size of the lesion and topoisomerase II alpha positivity (p=0.700). O’Malley FP et al [10] in their study showed a statistical significance of (p=0.04). In contrary to the above study, Press MF et al [11] in their study have not found any statistical correlation of the size of the lesion and topoisomerase II alpha expression, similar to our study.

In our 65 cases of invasive breast carcinoma, 58 were invasive ductal carcinoma, six were invasive lobular carcinoma and one mucinous carcinoma. Invasive ductal carcinoma showed a significant correlation with topoisomerase II alpha (p=0.021) in concordance with Mrklić I et al [7] which is a study in 83 cases of invasive ductal carcinoma. In our study, invasive lobular carcinoma also showed a significant statistical correlation similar to Arriola E et al [12].

In our study, Grade III tumours according to Nottingham histological grade were commonly observed. No statistical significance was seen in all the grades. But Koren R et al [13], in their study of 50 cases of invasive carcinoma breast, have shown a significant correlation in higher grade tumour versus topoisomerase II alpha positivity.

In our study, nodal positivity in invasive breast cancer did not show any statistical correlation with topoisomerase II alpha expression (p=0.981), whereas Depowski PL et al [5] in a study of 184 patients have shown a statistical significance between topoisomerase II alpha and nodal metastasis. In contrary to this, El Rebey HS et al [6] in his study showed no significant correlation.

In a study by Hellemans P et al [14] in 65 cases of invasive breast carcinoma 29% of the cases with distant metastasis showed immune reactivity with topoisomerase II alpha with a significant correlation value of p=0.005. In our study, only 3% of cases showed metastasis, hence no significant correlation was achieved.

Among the 65 cases of invasive breast carcinoma, ER/PR positivity was observed in 36% of cases that were wopoisomerase II alpha positive. No statistical significance was observed (p=0.267). According to Depowski PL et al [5], no statistical significance was observed similar to our study. Similarly, even Fountzilas G et al [15] in their study also showed no statistical significance. 

Kalogeraki A et al [16] in a study of 50 patients in a fine needle aspiration biopsy (FNAB) sample found a statistically significant correlation between topoisomerase II alpha and HER2/neu expression with p=0.005. Similarly, Depowski PL et al [5] also showed a significant value of p=0.001 between topoisomerase II alpha and HER2/neu. In our study, 64.7% of cases showed topoisomerase II alpha positivity with a significant statistical correlation (p=0.001).

In our study, among the 14 cases of Luminal A type of breast cancer, only one showed positivity for topoisomerase II alpha; this was found significant (p=0.001). A similar observation as seen by Romero A et al [17] in a study conducted in 61 cases.

Among the 11 cases of Luminal B type of breast cancer observed, eight cases showed positivity for topoisomerase II alpha with a significance value p=0.040. This was in concordance with Romero A et al [17] study who also found significant expression of topoisomerase II alpha in Luminal type B invasive breast cancer. 

In our study, 35.7% of cases of the triple-negative type of breast cancer showed topoisomerase II alpha positivity with no significant statistical correlation (p=0.446). On the contrary, a study conducted by Tan DS et al [8] showed a significant statistical correlation of p=0.01.

As already discussed above, in our study, HER2/neu molecular type of breast cancer shows a significant statistical correlation of p=0.048. Orlando L et al [18] in their study of 286 patients showed a significant association of topoisomerase II alpha and HER2/neu type of mammary cancer. They also went on to prove that topoisomerase II alpha expression in HER2/neu amplified patients increases the sensitivity of anthracycline-based chemotherapy.

With the above discussion on the molecular type of breast cancer versus topoisomerase II alpha expression according to Romero A et al [17], high-proliferative subtypes, such as Basal-like, Luminal B, and HER2-enriched, expressed higher levels of topoisomerase II alpha than Luminal A.

## Conclusions

Top 2 A expression is seen in invasive ductal carcinoma and invasive lobular carcinoma. There is no definite correlation between Top 2 A expression and tumour size, tumour grade, axillary lymph node metastasis, carcinoma in situ changes and lymphovascular invasion. High proliferative molecular subtypes like Luminal A, Luminal B and HER2/neu shows a significant correlation with Top 2 A. HER2/neu positivity suggests a strong relation with Top 2 A expression, hence proposes a high grade malignancy. Further studies are necessary to determine the exact prognostic value of this enzyme in breast cancer.
